# Herpes Simplex Virus type-1 infection induces synaptic dysfunction in cultured cortical neurons via GSK-3 activation and intraneuronal amyloid-β protein accumulation

**DOI:** 10.1038/srep15444

**Published:** 2015-10-21

**Authors:** Roberto Piacentini, Domenica Donatella Li Puma, Cristian Ripoli, Maria Elena Marcocci, Giovanna De Chiara, Enrico Garaci, Anna Teresa Palamara, Claudio Grassi

**Affiliations:** 1 Institute of Human Physiology, Medical School, Università Cattolica, 00168 Rome, Italy; 2 Department of Public Health and Infectious Diseases, Sapienza University of Rome, 00185 Rome, Italy; 3 Institute of Translational Pharmacology, National Research Council, 00133 Rome, Italy; 4 San Raffaele Pisana Scientific Institute for Research, Hospitalization and Health Care, 00163 Rome, Italy

## Abstract

Increasing evidence suggests that recurrent Herpes Simplex Virus type 1 (HSV-1) infection spreading to the CNS is a risk factor for Alzheimer’s Disease (AD) but the underlying mechanisms have not been fully elucidated yet. Here we demonstrate that in cultured mouse cortical neurons HSV-1 induced Ca^2+^-dependent activation of glycogen synthase kinase (GSK)-3. This event was critical for the HSV-1-dependent phosphorylation of amyloid precursor protein (APP) at Thr668 and the following intraneuronal accumulation of amyloid-β protein (Aβ). HSV-1-infected neurons also exhibited: i) significantly reduced expression of the presynaptic proteins synapsin-1 and synaptophysin; ii) depressed synaptic transmission. These effects depended on GSK-3 activation and intraneuronal accumulation of Aβ. In fact, either the selective GSK-3 inhibitor, SB216763, or a specific antibody recognizing Aβ (4G8) significantly counteracted the effects induced by HSV-1 at the synaptic level. Moreover, in neurons derived from APP KO mice and infected with HSV-1 Aβ accumulation was not found and synaptic protein expression was only slightly reduced when compared to wild-type infected neurons. These data further support our contention that HSV-1 infections spreading to the CNS may contribute to AD phenotype.

Alzheimer’s disease (AD) is a neurodegenerative disorder characterized by progressive decline in cognitive functions leading to memory loss and dementia^1^. Among the numerous factors concurring to AD pathogenesis, infectious agents may play a key role. In particular, epidemiological and experimental evidence demonstrated a link between HSV-1 infection reactivation and AD (references in 2, 3, 4). We previously reported that HSV-1 binding to the plasma membrane of rat cortical neurons induces membrane depolarization due to activation of persistent Na^+^ currents and inhibition of “leak” K^+^ currents that, in turn, triggers intracellular Ca^2+^ signals leading to increased intracellular Ca^2+^ levels ([Ca^2+^]_i_)^5^. These signals trigger phosphorylation of threonine at position 668 (Thr668) of amyloid precursor protein (APP) as well as of the same amino acid residue in the C-terminus fragment of APP, named AID or AICD^6^,^7^.

Following HSV-1-induced phosphorylation at Thr668, APP is subjected to multiple cleavages by secretases (β and γ) and caspases. As a consequence, several APP fragments (APPFs) including amyloid-β protein (Aβ) are formed and they are released in the extracellular medium and accumulated intracellularly^5^,^8^. It is known that Thr668 of APP and its C-terminus fragment are substrate for the glycogen synthase kinase (GSK)-3, as well as for several other kinases including c-Jun N-terminus kinase (JNK) and Cyclin-dependent kinase 5 (Cdk-5)^9^,^10^,^11^,^12^. GSK-3 is a Ser/Thr kinase composed of two isoforms (α and β) that plays a central role in AD^13^. GSK-3 is markedly activated in the brains of AD mouse models^14^,^15^ and patients^16^. This activation, obtained by either increased phosphorylation at Tyr residues (279 or 216 for α and β isoforms, respectively) or decreased phosphorylation at Ser sites (21α and 9β), has been reported to mediate synaptic damage induced by Aβ^17^. On the other hand, reduced activation of GSK-3β results in decreased APP processing and lower production and intracellular accumulation of Aβ^18^. Intraneuronal Aβ accumulation causes numerous structural and functional synaptic alterations including reduced expression of presynaptic proteins, and impaired synaptic transmission and plasticity^19^,^20^,^21^,^22^,^23^,^24^. Notably, function and structure of synapses also depend on GSK-3 activation^17^,^25^,^26^,^27^.

Although many papers including ours suggested the involvement of HSV-1 infection in AD pathogenesis, data demonstrating the effects of HSV-1 on the synaptic function underlying learning and memory are still lacking. Here we report that HSV-1 infection markedly affects synaptic function via GSK-3-dependent intraneuronal accumulation of Aβ.

## Results

### Ca^2+^-mediated activation of GSK-3 induced by HSV-1 is critical for APP phosphorylation at Thr668

We previously demonstrated that HSV-1 infection induces Ca^2+^-dependent phosphorylation of APP at Thr668 (pAPP) in rat cortical neurons *in vitro*^5^. Immunoreactivity for pAPP was found on the membrane and in the nuclei of infected cells, thus suggesting that the antibody raised against pAPP also recognized the C-terminus fragment of APP, AICD, that accumulates in the nucleus and is phosphorylated at the same threonine residue^6^.

**Figure 1:**
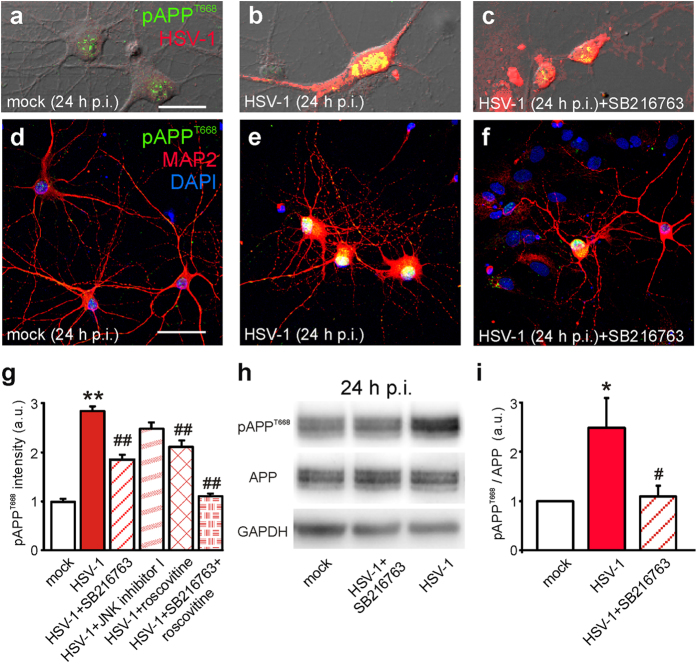
HSV-1-induced APP phosphorylation at Thr668 depends on GSK-3 and Cdk-5 activation. (**a**,**b**,**d,e**) Representative examples of pAPP^T668^ immunoreactivity (green) in mock- and HSV-1-infected neurons at 24 h p.i., respectively. Phosphorylation of APP markedly increased in HSV-1-treated neurons. (**c**,**f**) The presence of the selective GSK-3 inhibitor SB216763 (10 μM) in the culture medium significantly reduced (−54%) the increase in pAPP immunoreactivity observed in HSV-1-infected neurons. Red staining indicates HSV-1 and MAP2 immunoreactivity in (**a**–**f**), respectively. (**g**) Bar graphs showing mean pAPP fluorescence intensity in the conditions represented in a-c (mock, HSV-1 and HSV-1+SB216763) and in HSV-1-infected cells in the presence of inhibitors of JNK and Cdk-5 (JNK inhibitor I and roscovitine, respectively). Combination of SB216763 and roscovitine prevented the effects of HSV-1 on pAPP immunoreactivity. (**h**) Western blot analysis of pAPP^T668^, APP and GAPDH carried out on lysates of mock-infected cells, HSV-1-infected cells at 24 h p.i., and infected cells treated with the specific inhibitor of GSK-3 SB216763 for all the time p.i. (**i**) Densitometric analysis of three independent Western blot experiments. Scale bar: 20 μm in panels (**a**–**c**) and 50 μm in panels (**d-f**). *P < 0.05 *vs.* mock; **P < 0.001 *vs.* mock; #P < 0.05 *vs.* HSV-1; ##P < 0.001 *vs.* HSV-1. In this, and the following figures, statistical significance was assessed by Student’s *t* test for comparison between mock and HSV-1, and by one-way ANOVA with Bonferroni post-hoc test for multiple comparisons. For experiments that included fewer than 10 observations (e.g. densitometric analysis of WB data), the Mann-Whitney (Wilcoxon) statistic was used.

Given that APP is substrate for several kinases including GSK-3, JNK and Cdk-5, we studied their involvement in HSV-1-induced APP phosphorylation by adding specific kinase inhibitors to the culture medium of infected cells during the entire post-infection (p.i.) time. Twenty-four hours p.i. pAPP immunoreactivity was 1.85 ± 0.08 times larger than that found in mock-infected cells (*n* = 131 *vs*. 125, respectively; P = 1.3 × 10^−57^, assessed by Student’s *t* test; Fig. 1a,b,d,e,g). One way ANOVA revealed statistically significant differences after kinase inhibitor application (F_4,413_ = 10.2; P = 1.1 × 10^−11^). In particular, the ATP-competitive GSK-3 inhibitor, SB216763 (10 μM; Tocris, Minneapolis, MN) reduced the HSV-1-induced increment in pAPP immunoreactivity by 54% (from 1.85 ± 0.08 to 0.86 ± 0.10; *n* = 65; P = 9.9 × 10^−15^ *vs*. HSV-1 with no inhibitors, assessed by Bonferroni post-hoc test; Fig. 1c,f,g). Western blot data confirmed these results (Fig. 1h,i). The inhibitor of Cdk-5, roscovitine (10 μM, Sigma; St. Louis, MO) reduced the HSV-1-dependent increase in pAPP by 39% (from 1.85 ± 0.08 to 1.12 ± 0.11; *n* = 40; P = 3.9 × 10^−7^ *vs*. HSV-1 alone; Fig. 1g) whereas the JNK inhibitor “I” (5 μM, Merck Millipore, Billerica, MA) had a slight but not significant effect (the fluorescence increase was 1.49 ± 0.12; *n* = 56; P = 0.49 *vs*. HSV-1 alone; Fig. 1g). When SB216763 and roscovitine were applied together HSV-1 failed to significantly increase pAPP immunoreactivity (P = 6.5 × 10^−2^, Student’s *t* test, mock *vs*. HSV-1+inhibitors; *n* = 125 and 126, respectively; Fig. 1g). These data suggest that both GSK-3 and Cdk-5 are involved in the HSV-1-induced phosphorylation of APP, with the former playing a prominent role.

**Figure 2:**
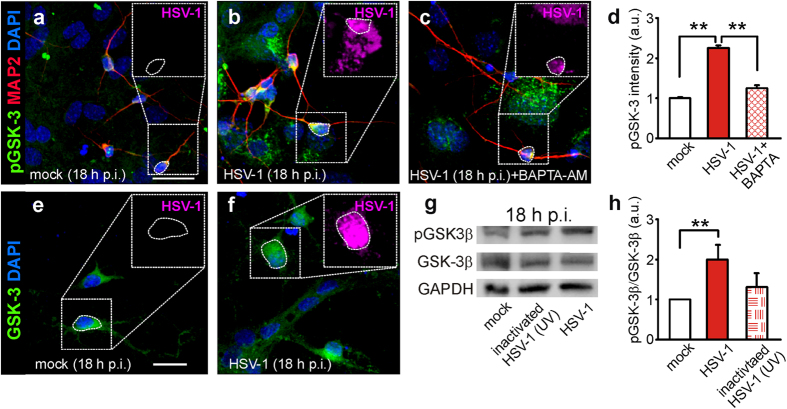
HSV-1 induces Ca^2+^-dependent activation of GSK-3. (**a**–**c**) Representative examples of MAP2^+^ neurons (red) immunoreactive for GSK-3α/β phosphorylated at Tyr279/216 (pGSK-3; green), in mock- (a), HSV-1-infected neurons at 18 h p.i. (**b**), and infected neurons exposed to the intracellular Ca^2+^ chelator BAPTA-AM (1 μM) for all the time p.i. (**c**). HSV-1 immunoreactivity (magenta) is shown in the dotted boxes inside the panels. The presence of BAPTA-AM (1 μM) significantly reduced the effects of HSV-1 on pGSK-3 immunoreactivity. (**d**) Bar graphs showing the mean pGSK-3 fluorescence intensity in the conditions represented in (**a**–**c**). (**e**,**f**) Representative examples of mock- and HSV-1-infected neurons immunostained for GSK-3 (green) and HSV-1 (magenta) at 18 h p.i. No significant changes were observed. (**g**) Western blot analysis of pGSK-3β^Y216^, total GSK and GAPDH carried out on lysates of mock-infected cells, HSV-1-infected cells at 18 h p.i., and cells challenged for 1 h with UV-inactivated HSV-1 and then cultured for further 18 hours. (**h**) Densitometric analysis of three independent Western blot experiments. Scale bars: 50 μm. **P < 0.001.

Therefore, we further investigated the effects of HSV-1 on GSK-3 activation by studying its phosphorylation at Tyr279 and 216 (pGSK-3) that are well known activatory sites for α and β isoforms of this kinase, respectively. At 18 h p.i. pGSK-3 immunoreactivity was 1.25 ± 0.07 times greater than that of mock-infected neurons (*n* = 71 and 244, respectively; P = 1.3 × 10^−71^, Student’s *t* test; Fig. 2a,b,d). This effect, that preceded the marked increase in APP phosphorylation we observed at 24 h p.i., was counteracted by SB216763 (10 μM), thus confirming the specificity of pGSK-3 immunostaining (data not shown).

In previous studies, we demonstrated that HSV-1 binding to the cell membrane triggered intracellular Ca^2+^ signals producing APP phosphorylation at Thr668^5^. These Ca^2+^ transients depended on both Ca^2+^ entry through Ca_v_1 channels and Ca^2+^ release from intracellular stores, eventually leading to [Ca^2+^]_i_ increases^5^. Based on these findings, we asked whether Ca^2+^ signals regulate GSK-3 phosphorylation at Tyr279/216 upstream APP phosphorylation. In the presence of the intracellular Ca^2+^ chelator BAPTA-AM (1 μM, Sigma), the HSV-1-induced increase in pGSK-3 immunoreactivity was reduced by 80% at 18 h p.i. (from 1.25 ± 0.07 to 0.25 ± 0.07; *n* = 40, P = 2.3 × 10^−7^ *vs*. HSV-1, Student’s *t* test ; Fig. 2c,d) thus indicating that intracellular Ca^2+^ dyshomeostasis triggered by HSV-1 is involved in the Tyr-dependent activation of GSK-3.

Given that many studies pointed out the importance of the GSK-3β isoform in mediating the action of this kinase, we performed Western blot experiments to determine if GSK-3β was activated following HSV-1 infection. Notably, we found a marked increase of pGSK-3β in HSV-1-infected neurons (Fig. 2g,h) whereas the total expression of GSK-3β was not significantly modified, as also demonstrated by immunofluorescence (total GSK-3 immunoreactivity was: 1.00 ± 0.06 and 1.09 ± 0.07 in mock and HSV-1-infected cells, *n* = 22 and 28 respectively; P = 0.07 Student’s *t* test; Fig. 2e–h). Finally, to determine whether the observed effects depended on HSV-1 binding to the cell membrane, we repeated Western blot experiments in cells treated with UV-inactivated HSV-1 that retains the ability to bind the membrane and activate the signaling cascade leading to increased [Ca^2+^]_i_ but it does not replicate inside cells. No significant increases in pGSK-3β/GSK-3β ratio were induced by UV-inactivated HSV-1 (P = 0.10 *vs*. mock; Fig. 2g,h).

In agreement with our recent findings in rat cortical neurons^28^ and other literature reports^29^,^30^, HSV-1 infection also slightly increased GSK phosphorylation at Ser9 (Suppl. Fig. 1). However, the effect of the GSK-3 inhibitor SB216763 on APP phosphorylation suggests that the “net” result of HSV-1-induced GSK-3 phosphorylation is the kinase activation.

### GSK-3 activation mediates intraneuronal accumulation of Aβ

**Figure 3:**
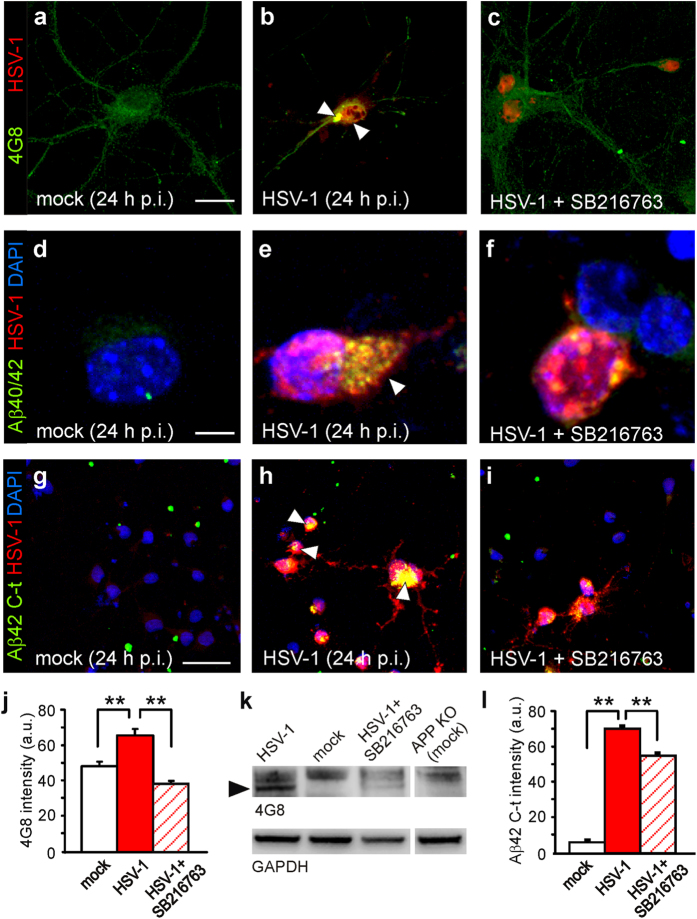
GSK-3 activation is involved in HSV-1-induced Aβ production and accumulation. Representative examples of mock-infected, HSV-1-infected and HSV-1-infected cortical neurons in the presence of SB216763, immunostained for HSV-1 and 4G8 (**a**–**c**), or a specific antibody against Aβ40/42 (**d**–**f**). (**g**–**i**) Representative examples of cortical neurons in the same conditions shown in (**a**–**f**) immunostained with antibodies specifically recognizig Aβ42 C-terminus and HSV-1. Arrow heads indicate Aβ accumulation. DAPI was used to label cell nuclei. (**j**,**l**) Bar graphs quantifying 4G8 and Aβ42 C-t immunoreactivity in HSV-1-positive neurons under the conditions shown in (**a**–**c**) and (**g**–**i**), respectively. (**k**) Western blot analysis performed using 4G8 antibody on lysates of mock-infected cells, HSV-1-infected cells at 24 h p.i. in the presence or not of SB216763, and mock-infected APP KO cells that were used as negative control. Black arrow head indicates a band at molecular weight of about 35 kDa. Scale bars: 20 μm in panels (**a**–**c**); 10 μm in panels d-f and 50 μm for panels (**g**–**i**). **P < 0.001.

We previously demonstrated that the culture medium of HSV-1-infected neurons contained small soluble Aβ oligomers (i.e., dimers and trimers), and lysates of infected neuronal cells subjected to Western blotting exhibited a band recognized by the 4G8 antibody (that specifically reacts with murine high molecular weight Aβ aggregates) at 35 kDa, likely corresponding to a Aβ nonamer^5^,^8^. Here we showed that mouse cortical neurons infected with HSV-1 exhibited an increased immunoreactivity for Aβ40/42 with respect to mock-infected cells (Fig. 3a,b,d,e). We then specifically looked for intracellular accumulation of Aβ42 in infected neurons, that was assessed by an antibody recognizing the C-terminus region of the protein (Fig. 3g,h). In agreement with our previous reports, the specificity of Aβ42 C-t immunolabeling was assessed by adding β- and γ-secretase inhibitors to the culture medium during the time p.i.^5^,^8^. Under these experimental conditions Aβ42 C-t immunoreactivity was significantly reduced (data not shown).

Here we found that inhibition of GSK-3 activity by SB216763 significantly reduced Aβ fluorescence intensity in HSV-1-immunoreactive neurons. In fact, the increase in 4G8 immunoreactivity was completely abolished (P = 7.2 × 10^−12^; Student’s *t* test; *n* = 40 for HSV-1 and *n* = 38 for HSV-1+SB216763; Fig. 3b,c,j) whereas immunolabeling with the Aβ42 C-t antibody was reduced by 23% of untreated infected neurons (P = 2.8 × 10^−4^, Student’s *t* test; *n* = 43 for both mock and HSV-1-infected cells; Fig. 3h,i,l). These data indicate that HSV-1-induced Aβ production and accumulation depend on GSK-3 activation. Western blot experiments confirmed these data on the band recognized by 4G8 at 35 kDa in lysates from infected cells (Fig. 3k).

Notably, cell treatment with SB216763 added after virus adsorption did not affect HSV-1 infection (viral titer: 6.4 × 10^3^ *vs.* 5.4 × 10^3^ pfu/mL without inhibitor, at 24 h p.i.).

### Expression of the presynaptic proteins synaptophysin and synapsin-1 is affected by HSV-1 infection

**Figure 4:**
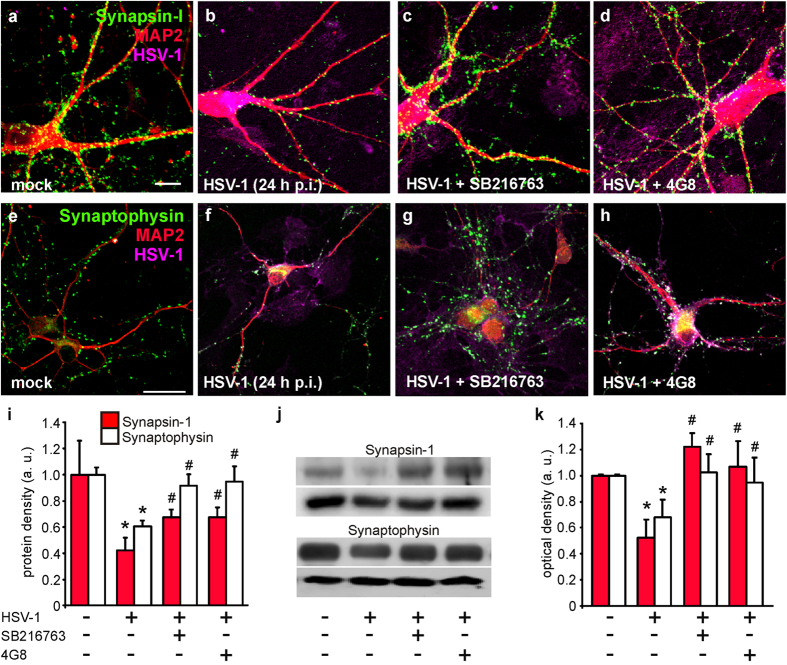
HSV-1 infection induces Aβ- and GSK-3-dependent reduction of synaptic protein expression. Mouse cortical neurons (DIV 15) were infected with HSV-1 in the presence or not of either SB216763 (10 μM) or 4G8 antibody (3.3 μg/mL). **(a**–**h**) show representative examples of mouse cortical neurons immunostained for synapsin-1, MAP2 and HSV-1 or synaptophysin, MAP2 and HSV-1, respectively, at 24 h p.i. (**i**) Bar graphs quantifying synapsin-1 and synaptophysin density (see methods). (**j**) Western blot analysis of synapsin-1 and synaptophysin performed on lysates of mock-infected cells, HSV-1-infected cells at 24 h p.i., and cells treated with SB216763 or 4G8 antibody. GAPDH and tubulin were used as loading control for synapsin-1 and synaptophysin respectively. (**k**) Densitometric analysis of three independent Western blot experiments. Scale bars: 10 μm and 30 μm for panels (**a**–**h**), respectively. *P < 0.001 *vs.* mock; #P < 0.005 *vs.* HSV-1.

It is known that intracellular Aβ accumulation affects synaptic function^21^,^23^,^24^. Functional and structural alterations of synapses may also depend on GSK-3 activation^17^,^25^,^27^. Therefore, we investigated the effects of HSV-1 infection on: i) the expression of the presynaptic proteins synapsin-1 and synaptophysin; and ii) synaptic transmission *in vitro*, to subsequently determine the role of GSK-3 activation and Aβ accumulation in these effects. Twenty-four h p.i. we found a significant reduction in the expression of both proteins: −58% respect to mock-infected neurons for synapsin-1 (*n* = 14 and 12 fields, P = 1.5 × 10^−3^, Student’s *t* test; Fig. 4a,b,i); −39% for synaptophysin (*n* = 21 and 19 microscopic fields [at 63 ×  magnification] analyzed for mock- and HSV-1-infected cells, respectively; P = 1.5 × 10^−7^, Student’s *t* test; Fig. 4e,f,i). These effects were clearly due to HSV-1 infection. In fact, when neuronal cultures were treated with UV-inactivated virus no alterations in protein expression were observed at 24 h p.i. (*n* = 18 and 16 fields analyzed, P = 0.99 *vs*. uninfected cells, Student’s *t* test). The effects of HSV-1 infection on presynaptic proteins clearly depended on GSK-3 activation and Aβ accumulation (one-way ANOVA: F_2,35_ = 10.3, P = 2.9 × 10^−4^ for synaptophysin and F_2,37_ = 9.0, P = 6.5 × 10^−4^ for synapsin-1). In fact, when neurons were treated with the GSK-3 inhibitor SB216763 (10 μM) the inhibitory effects of HSV-1 on synapsin-1 expression were significantly smaller than in infected, untreated neurons and the effects on synaptophysin expression were almost completely abolished. In particular, synapsin-1 density increased from 0.42 ± 0.10 to 0.68 ± 0.6 (*n* = 14 fields analyzed, P = 1.8 × 10^−3^, Bonferroni post-hoc test; Fig. 4b,c,i), whereas synaptophysin density increased from 0.61 ± 0.04 to 0.95 ± 0.12, with the mock-infected condition set at 1 (*n* = 10, P = 2.0 × 10^−3^, Bonferroni post-hoc test; Fig. 4f,g,i).

In agreement with previous reports^31^, addition of the GSK-3 inhibitor to the culture medium of mock-infected neurons significantly increased the expression of synapsin-1 (+36% of mock-infected neurons without SB216763; *n* = 13 microscopic fields; P = 7.3 × 10^−3^; Student’s *t* test), but it did not affect synaptophysin expression (*n* = 12 microscopic fields; P = 0.67; Student’s *t* test).

**Figure 5:**
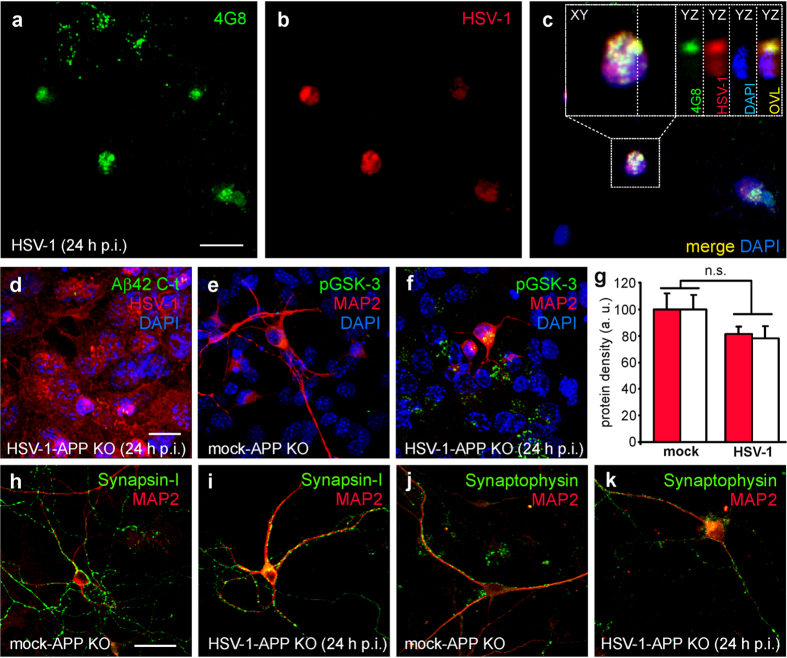
Extracellularly applied 4G8 antibody is internalized in wild-type infected neurons and recognizes Aβ produced following infection whereas HSV-1-infected APP KO cortical neurons exhibits neither Aβ42 accumulation nor reduction of synaptic proteins. (**a**–**c**) Representative examples of 4G8 antibody internalization in neurons infected with HSV-1 for 24 hours. 4G8 antibody was added to the culture medium for all the time p.i. and, after its internalization, it recognized intracellular Aβ produced following HSV-1 infection. 4G8 was revealed with secondary antibody after fixation and permeabilization of 4G8-treated cells at 24 h p.i. High magnification image of cell boxed in panel c shows that 4G8 immunostaining was localized outer the nuclei of infected (HSV-1-positive) cells. Cell nuclei were stained with DAPI and are displayed in blue. (**d**) Representative example of HSV-1-infected APP-KO mouse cortical neurons and astrocytes at 24 h p.i. immunostained for Aβ42, HSV-1 and DAPI. No Aβ42 immunoreactivity was observed. (**e**,**f**) Infected APP-KO neurons (MAP2^+^, red) exhibited marked increase in pGSK-3 staining (green). (**g**) Bar graph quantifying synapsin-1 (red) and synaptophysin (white) densities in the conditions represented in (**h**–**k**). (**h**–**k**) Representative examples of synapsin-1 and synaptophysin staining of mock- and HSV-1-infected APP-KO cortical neurons (DIV 15) at 24 h p.i. Scale bars: 20 μm for panels (**a**–**f**); 30 μm for panels (**h**–**k**).

Finally, we determined if the intracellular Aβ accumulation that we observed following infection was involved in the GSK-3-dependent reduction of synaptic protein expression. To address this issue, we exposed neuronal cultures to 4G8 antibody (3.3 μg/mL) during the post-infection period. It is known that extracellularly-applied 4G8 interacts with the extracellular site of APP, it is internalized and accumulated intracellularly^20^. Once inside neurons, 4G8 antibody may bind Aβ and counteract its effects. In our experimental conditions, extracellular 4G8 was indeed internalized and it recognized HSV-1-produced Aβ (Fig. 5a–c). We found that the expression of synapsin-1 and synaptophysin in HSV-1-infected neurons (24 h p.i.) exposed to 4G8 was significantly different from that of HSV-1-infected cells. In the presence of 4G8 the reduction of synapsin-1 and synaptophysin immunoreactivity passed from −58% to −33% and from –39% to –5%, respectively (P = 3.3 × 10^−3^ and P = 3.2 × 10^−3^, respectively *vs*. HSV-1; Bonferroni post-hoc test; Fig. 4d,h,i). These findings were supported by results of Western blot analyses (Fig. 4j,k).

To further investigate the dependence of the HSV-1-induced synaptic effects on APPF production, we infected cortical neurons isolated from APP-KO mice. Given that these cells do not express APP, HSV-1 failed to produce intracellular accumulation of Aβ42 (compare Fig. 5d with Fig. 3h) although APP-KO neurons exhibited a clear HSV-1-induced activation of GSK-3 (Fig. 5e,f). When the immunolabeling of synaptophysin and synapsin-1 was evaluated in APP-KO-infected neurons, we found slight but not significant differences respect to APP-KO-mock-infected cells (for synapsin-1: P = 0.12, Student’s *t* test; *n* = 13 and 11 fields analyzed for mock and HSV-1-infected cells, respectively, Fig. 5h,i,g; for synaptophysin: P = 0.12, Student’s *t* test; *n* = 9 and 14 fields analyzed for mock and HSV-1-infected cells, respectively, Fig. 5j,k,g), thus supporting our contention that HSV-1-induced Aβ accumulation is responsible for synaptic protein downregulation.

### Synaptic transmission is affected by HSV-1 infection

**Figure 6:**
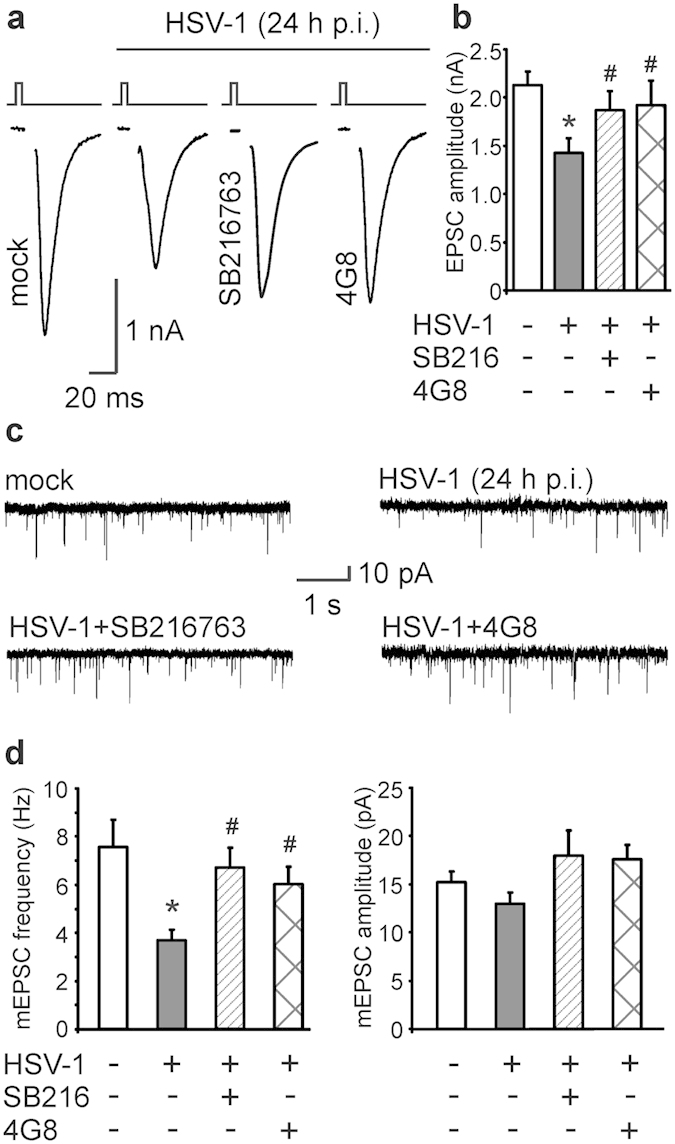
HSV-1 infection impairs synaptic transmission. (**a**) Representative examples of evoked excitatory post-synaptic currents (EPSCs) recorded in mock- and HSV-1-infected (24 h p.i.) autaptic neurons cultured with or without either SB216763 (10 μM) or 4G8 (3.3 μg/mL). (**b**) Bar graph quantifying the amplitude of EPSCs under the above described conditions. (**c**) Representative examples of spontaneous activity (miniature excitatory post-synaptic currents, mEPSCs) recorded in autaptic neurons infected or not with HSV-1 (24 h p.i.) and cultured with or without either SB216763 or 4G8. (**d**) Bar graph showing the mean frequency (left) and amplitude (right) of mEPSCs. *P < 0.005 *vs.* mock; #P < 0.05 *vs.* HSV-1.

We then evaluated whether the molecular alterations induced by HSV-1 at the presynaptic level impaired synaptic transmission. To address this issue we studied the amplitude of evoked excitatory post-synaptic currents (EPSCs) and the amplitude and the frequency of spontaneous miniature EPSCs (mEPCS). When autaptic cultures of mouse cortical neurons^23^,^24^ were infected with HSV-1 for 24 hours, EPSC amplitude was significantly reduced if compared to that of mock-infected neurons (−36%; *n* = 10 and 19 for mock- and HSV-1-infected neurons, respectively; P = 1.7 × 10^−3^; Student’s *t* test; Fig. 6a,b). The same treatment also markedly reduced the frequency of mEPSC (−49% *vs.* mock-infected neurons; *n* = 10 for mock and 18 for HSV-1-infected neurons; P = 6.0 × 10^−4^; Student’s *t* test; Fig. 6c,d), whereas their mean amplitude was only slightly reduced (−15% *vs*. mock infected neurons, P = 0.15; Fig. 6d). The inhibitory effects of HSV-1 on both EPSC amplitude and mEPCS frequency were significantly reversed by the presence of 10 μM SB216763 in the culture medium, and they depended on Aβ accumulation. Indeed, according to what we observed for presynaptic protein expression, 4G8 rescued synaptic transmission from the inhibitory effects of HSV-1. In particular, no significant differences were found between EPSC amplitudes recorded in mock-infected neurons and in neurons infected with HSV-1 and treated with either SB216763 or 4G8 for all the time p.i. (one way ANOVA: F_2,28_ = 0.83, P = 0.44; Fig. 6a,b). Similar results were found for the mEPSCs frequency that was increased from 3.7 Hz in HSV-1-infected neurons to 6.7 Hz (*n* = 12) in the presence of the GSK-3 inhibitor and to 6.0 Hz (*n* = 10) in the presence of 4G8 antibody (P = 2.6 × 10^−3^ and P = 3.6 × 10^−2^, respectively; Bonferroni post-hoc test; Fig. 6c,d).

### HSV-1 inhibits CREB via GSK-3 and Aβ

**Figure 7:**
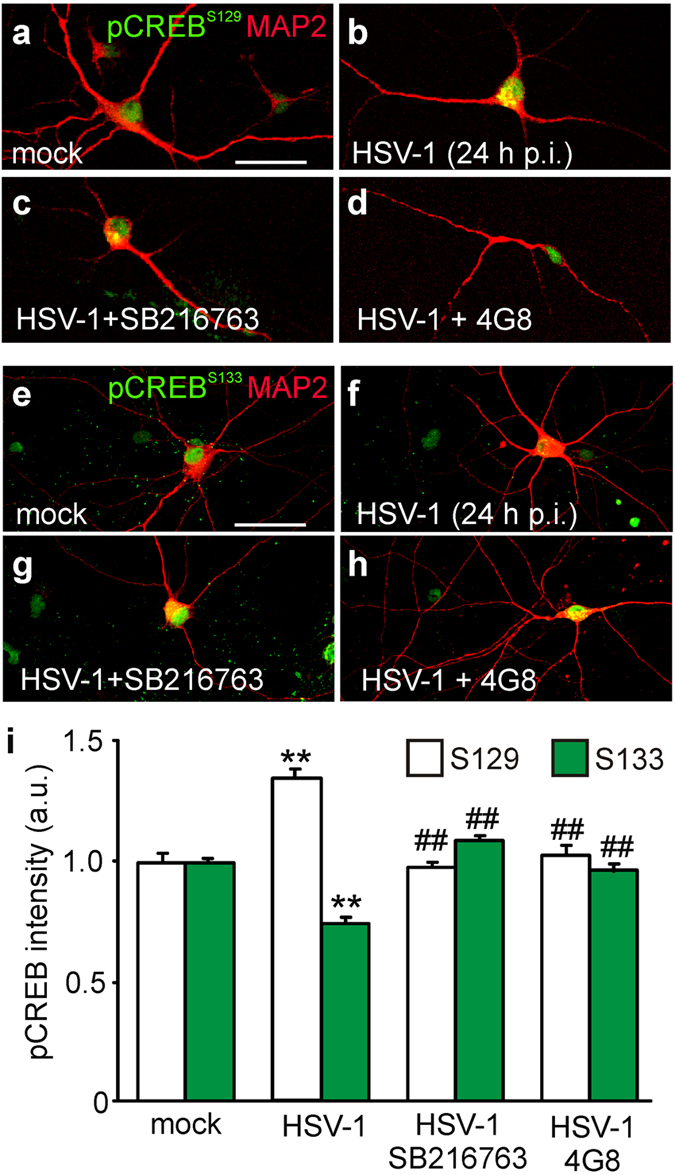
HSV-1 inhibits the transcription factor CREB via GSK-3 and Aβ. (**a**–**d**) Representative images of mock- and HSV-1-infected neurons at 24 h p.i. cultured with or without either SB216763 (10 μM) or 4G8 (3.3 μg/mL) and immunostained for pCREB^S129^ and MAP2. (**e**–**h**) Representative images of neurons cultured in the same conditions of (**a**–**d**) and immunostained for pCREB^S133^ and MAP2. (**i**) Bar graphs showing mean fluorescence intensities of CREB phosphorylation at Ser129 and Ser133, in the conditions represented in (**a**–**h**). Notably, the presence of SB261763 or 4G8 strongly counteracted the effect of HSV-1 on CREB phosphorylations. Scale bars: 30 μm. **P < 0.001 *vs.* mock; ##P < 0.001 *vs.* HSV-1.

Having demonstrated that HSV-1 infection induces GSK-3 activation and Aβ accumulation, we looked for downstream components of the HSV-1-triggered molecular pathway likely responsible for the synaptic dysfunction we observed. Among the numerous potential candidates, we focused on the cAMP response element-binding protein (CREB) because it is a critical determinant of synaptic function, it is target of Aβ and GSK-3β activation has been suggested to inhibit the CREB-mediated transcriptional program^17^,^32^. First, we determined whether CREB activity was modulated by HSV-1. CREB has two mutually exclusive phosphorylation sites that regulate its activity: Ser133 (activatory) and Ser129 (inhibitory), the latter being phosphorylated by GSK-3^33^,^34^. We found that in HSV-1-infected neurons (24 h p.i.) pCREB^S129^ immunoreactivity was significantly increased with respect to mock-infected neurons (+35%; *n* = 88 and 92, respectively; P = 5.5 × 10^−10^, Student’s *t* test; Fig. 7a,b,i). On the contrary, fluorescence intensity for pCREB^S133^ was reduced by −27% (*n* = 79 and 82; P = 6.3 × 10^−17^, Student’s *t* test; Fig. 7e,f,i). Application of either SB216763 or 4G8 significantly reversed the effects of HSV-1 on CREB phosphorylation (one-way ANOVA; for pCREB^S129^: F_2,191_ = 32.1; P = 2.0 × 10^−11^; for pCREB^S133^: F_2,223_ = 65.4, P = 1.7 × 10^−11^). In particular, when SB216763 was present in the culture medium of infected cells the immunoreactivity of pCREB^S129^ and pCREB^S133^ was restored to values similar to those observed in mock-infected cells (P = 4.4 × 10^−11^ and 3.0 × 10^−15^ *vs.* HSV-1 alone, Bonferroni post-hoc test, *n* = 54 and *n* = 64, for S129 and S133, respectively; Fig. 7c,g,i). Similar results were obtained with 4G8 (P = 3.0 × 10^−8^ and 4.6 × 10^−12^ *vs*. HSV-1 alone, Bonferroni post-hoc, *n* = 52 and *n* = 80, for S129 and S133, respectively; Fig. 7d,h,i).

Interestingly, application of SB216763 to mock-infected cells exerted a much smaller effect on CREB phorsphorylation. Indeed, immunoreactivities for pCREB^S129^ and pCREB^S133^ were modified by less than 10% by inhibition of GSK-3 activity (+9%; P = 2.9 × 10^−2^ for S129 and –9% P = 1.4 × 10^−3^, for S133, *n* = 104 and 53, respectively), thus suggesting that this kinase plays a critical role in CREB activity especially following infection.

## Discussion

In this study we demonstrated that HSV-1 infection impairs synaptic function in cultured mouse cortical neurons through GSK-3 activation and intracellular accumulation of Aβ. These events were responsible for the HSV-1-induced molecular and functional alterations we observed at the synaptic level consisting of: i) reduced expression of the presynaptic proteins synapsin-1 and synaptophysin; ii) depressed synaptic transmission. Finally, we found that Aβ- and GSK-3-mediated inhibition of CREB is likely involved in the HSV-1-induced synaptic effects.

In a previous report we demonstrated that HSV-1 binding to neuronal membrane of rat cortical neurons *in vitro* induces membrane depolarization due to activation of persistent Na^+^ currents and inhibition of leak K^+^ currents that, in turn, triggers intracellular Ca^2+^ signals (due to both Ca^2+^ entry through Ca_v_1 channels and Ca^2+^ release from IP_3_ receptors) leading to increased [Ca^2+^]_i_^5^. Ca^2+^ transients triggered by HSV-1 binding to the membrane were responsible for APP phosphorylation at Thr668 as well as for phosphorylation of the same amino acid in the C-terminus region of APP, named AID or AICD^7^. The former is believed to be a key event in APP processing and Aβ production^10^,^35^, whereas the latter enables AICD to interact with the Fe65 adapter protein^36^ and enter the nucleus, where it affects gene transcription and induces neurodegeneration^6^,^37^. Very recently, we also demonstrated that the HSV-1-induced nuclear accumulation of ACID regulates the expression of the Aβ clearance/degrading enzyme neprilysin and GSK-3^28^. Here we found that HSV-1 infection induces an intense activation of GSK-3, as revealed by immunoreactivity for phospho-GSK-3α/β (at Tyr279/216). Western blot experiments also indicated that after viral infection GSK-3β phosphorylation at Tyr216 was significantly increased whereas the total amount of the protein did not significantly change, thus suggesting the specific activation of this kinase rather than upregulation of its expression following infection. HSV-1 infection also slightly increased Ser9 phosphorylation in mouse cortical neurons. In cultured human fibroblasts Naghavi and colleagues demonstrated that the viral Ser/Thr kinase Us3 inhibits GSK-3β activity by increasing its phosphorylation at Ser9^29^. However, in our experimental model, the “net” resulting activity of GSK-3 following 18–24 h of infection was increased. Indeed, the specific GSK-3 inhibitor, SB216763, reduced the HSV-1-induced increase in pAPP immunoreactivity by more than 50%. SB216763 has been reported to inhibit GSK-3 by specifically reducing pTyr279/216 rather than increasing pSer21/9^38^. Our experiments performed with the intracellular Ca^2+^ chelator BAPTA-AM indicate that intracellular Ca^2+^ increases observed several h p.i. are involved in GSK-3 activation and the subsequent phosphorylation of APP and AICD we observed. These results are in line with those of Hartigan and Johnson^39^ demonstrating that, in response to transient [Ca^2+^]_i_ increases induced by the ionophore A23187, GSK-3β activity was enhanced because of Tyr216 phosphorylation. Pierrot *et al.*^10^ also demonstrated that under depolarizing conditions GSK-3β phosphorylation at Try216 was increased in rat cortical neurons.

Our data also indicate that GSK-3 is involved in the HSV-1-induced production and intracellular accumulation of APPFs including Aβ40 and 42. Indeed, neuronal immunostainings with either antibodies labeling all APPFs containing the Aβ sequence 17–24 (i.e., anti-Aβ40/42 and 4G8) or the Aβ42 C-terminus antibody, were significantly reduced in HSV-1-infected cells treated with SB216763 during the p.i. period, and similar results were also obtained with Western blot experiments. The involvement of GSK-3 in Aβ production is fully supported by previous literature reports showing that *in vitro*: i) GSK-3 interacts with presenilins^15^; ii) GSK-3β promotes the production and intracellular accumulation of Aβ by enhancing pAPP^10^ as well as β-secretase activity^40^. Conflicting results have been reported *in vivo* where neither GSK-3α nor GSK-3β were found to modulate APP processing^41^, and GSK-3 inhibition reduced Aβ levels independently of APP processing modulation by acting on Aβ degradation/sequestration^42^.

Our findings provide novel evidence that HSV-1 infection affects synaptic function. To our knowledge this is the first paper documenting that in HSV-1-infected neurons there is a GSK-3/Aβ-dependent decrease in the expression of presynaptic proteins that is associated with depressed synaptic transmission.

The role of intracellular Aβ accumulation in synaptic function dysregulation underlying cognitive impairment is well known^19^,^20^,^21^,^22^,^23^,^24^. Here we demonstrated that using two different approaches counteracting or preventing HSV-1-induced Aβ production and accumulation (i.e., intracellular loading of 4G8 antibody and APP KO mice, respectively), HSV-1 lost its synaptotoxicity. The residual effects of HSV-1 that we found at the synaptic level under these experimental conditions might depend on several factors. First, 4G8 might be unable to fully counteract the APPFs produced following HSV-1 infection. In fact, Tampellini *et al.*^20^ showed that 24-h lasting 4G8 treatment of primary neurons from Tg2576 mice reduced intracellular Aβ levels by 30–40% only. We also found a slight reduction of presynaptic protein expression in HSV-1-infected APP null neurons. These findings allow us to speculate that other GSK-3-dependent and Aβ-independent mechanisms compromising synaptic function might play a minor role in HSV-1 effects. Increased GSK-3 expression has been shown to negatively modulate neurotransmitter release via phosphorylation of VGCCs^43^, and GSK-3 inhibition has been found to increase neurotransmission^44^ and synapsin-1 expression^31^. GSK-3 has been also found to be involved in the modulation of other ion channels including voltage-gated Na^+^ channels that may influence neuronal firing and synaptic transmission^45^,^46^,^47^,^48^,^49^. Moreover, GSK-3 up-regulation has been reported to impair synaptic plasticity at both functional and structural levels by reducing synapsin-1 clustering^50^ and dendritic spine density^17^, whereas GSK-3 inhibition by SB216763 or TDZD-8 reverted these effects. Finally, another possibility is that activated GSK-3 causes synaptic dysfunctions via Tau protein hyperphosphorylation^51^,^52^,^53^. All these data are in agreement with our finding that HSV-1 acts upstream GSK-3 activation and Aβ production. Aβ-independent effects due to virus binding to neuronal membrane did not play a role in the observed alterations of synaptic proteins. In fact, when neurons were infected with UV-pretreated virus retaining the ability to bind cell membrane but not that of replicating inside cells, no changes in synaptic protein expression were observed.

Our data also suggest that CREB is critically involved in the HSV-1-activated molecular pathway leading to synaptic dysfunction. CREB is a transcription factor whose activity is modulated by GSK-3β via phosphorylation at Ser129^33^,^34^,^54^. It also plays a pivotal role in structural and functional synaptic plasticity^17^,^55^,^56^. Here we found that CREB is inactivated following HSV-1 infection and that this inhibition depends on GSK-3 activation and Aβ accumulation. These findings allow us to hypothesize that GSK-3-induced CREB inhibition is a critical determinant of HSV-1-induced synaptic dysfunction although further studies are needed to gain in-depth understanding of its role in the observed effects.

Collectively, findings reported here suggest that HSV-1 alters the expression of synaptic proteins via activation of GSK-3 and intraneuronal accumulation of Aβ. Although the production and accumulation of Aβ has been reported in the temporal brain cortex of mice 5 days after intranasal inoculation with HSV-1^57^, further studies on wild-type and AD transgenic mice are needed to demonstrate the cause-and-effect relationships among recurrent HSV-1 infections, Aβ overproduction and synaptic dysfunction underlying cognitive impairment in AD.

## Material and Methods

### Primary cortical neuron cultures

Primary cultures of cortical neurons were obtained from E18 C57/bl6 mice and B6.129S7-App^tm1^Dbo/J mice as previously described^5^. Briefly, after removing brains, dissected cortices were incubated for 10 min at 37 °C in PBS containing trypsin-ethylenediaminetetraacetic acid (0.025%/0.01% w/v; Biochrom AG, Berlin, Germany), and the tissue was mechanically dissociated at room temperature (RT) with a fire-polished Pasteur pipette. The cell suspension was harvested and centrifuged at 235 × g for 8 min. The pellet was suspended in 88.8% Minimum Essential Medium (MEM, Biochrom), 5% fetal bovine serum, 5% horse serum, 1% glutamine (2 mM), 1% penicillin-streptomycin-neomycin antibiotic mixture (PSN, Invitrogen, Carlsbad, CA), and glucose (25 mM). Cells were plated at a density of 10^5^ cells on 20-mm coverslips (for immunocytochemical studies) and 10^6^ cells/well on 35-mm six-well plates (for Western blot studies), precoated with poly-L-lysine (0.1 mg/ml; Sigma). Twenty-four hours later, the culture medium was replaced with a mixture of 96.5% Neurobasal medium (Invitrogen), 2% B-27 (Invitrogen), 0.5% glutamine (2 mM), and 1% PSN. After 72 h, this medium was replaced with a glutamine-free version of the same medium, and the cells were grown for 10 more days before carrying-out experiments.

### HSV-1 infection

Infection was performed as previously described^5^. Fourteen days after plating, the culture medium was replaced with Neurobasal medium containing particles of HSV-1 strain F [5 × 10^8^ plaque forming units (PFU)/mL] at a multiplicity of infection (MOI) of 5, and neuronal cultures were incubated for 1 h at 37 °C (adsorption period). The HSV-1-containing medium was then removed and after 2 washes in PBS, the cells were returned to the original medium and cultured for 18–24 h in the presence or not of specific inhibitors left in the culture medium for all the time p.i. and then fixed in paraformaldehyde (PFA, 4% in PBS, Sigma). The supernatants of infected cells were then subjected to standard plaque assay^58^ to quantify virus production.

In all the experiments, the effects observed in infected cells were compared with those obtained in mock-infected controls that had been exposed to the vehicle alone. Additional control experiments were performed with virus that had been exposed on ice for 5 min to a 30 W, 254 nm, germicidal UV light placed at a distance of 15 cm (UV-inactivated HSV-1). UV-inactivated HSV-1 retains the capacity for plasma membrane binding and cell entry although it is incapable of replication^5^, as confirmed by standard plaque assay.

### Immunocytochemistry

Immunocytochemistry was performed as previously described^5^,^26^. Cortical neurons cultured for 15 DIV were infected with HSV-1 and then fixed with 4% PFA in PBS for 15 min at RT. After being permeabilized (10 min incubation with 0.3% Triton X-100 [Sigma] in PBS), the cells were incubated for 20 min with 0.3% bovine serum albumin in PBS to block nonspecific binding sites and then overnight at 4 °C with different pairs of the following antibodies: rabbit anti-APP phosphorylated at Thr668 (pAPP, 1:100; Cell Signalling Technology Inc., Danvers, MA); mouse anti-microtubule associated protein-2 (MAP2, 1:500; Immunological Sciences, Rome, Italy), rabbit anti-pGSK3 α/β (Tyr279/Tyr216) (1:300; Signalway Antibody, College Park, MD); rabbit anti-GSK-3β (1:300; Cell Signaling); goat anti-HSV-1 (1:500; Millipore); rabbit anti-Aβ42 C-terminus (1:300; Covance, Princeton, NJ); rabbit anti-Aβ40/42 AB5076 (1:300; Millipore); mouse anti-4G8 (1:300; Covance); rabbit anti-synaptophysin (1:300; Immunological Sciences); rabbit anti synapsin-1 XP (1:300; Cell Signaling); rabbit anti-CREB phosphorylated at Ser129 (1:500; Immunological Sciences); rabbit anti-CREB phosphorylated at Ser133 (1:500; Cell Signaling). The next day, cells were incubated for 90 min at RT with a mixture of the following secondary antibodies: Alexa Fluor 488 donkey anti-rabbit (1:1,000; Invitrogen); Alexa Fluor 633 donkey anti-goat (1:1,000; Invitrogen); Alexa Fluor 546 donkey anti-mouse (1:1,000; Invitrogen) Finally, nuclei were counterstained with 4′,6-diamidino-2-phenylindole (DAPI, 0.5 μg/ml for 10 min; Invitrogen), and the cells were coverslipped with ProLong Gold anti-fade reagent (Invitrogen).

Images (512 × 512 pixels) were acquired at 63 ×  magnification with a confocal laser scanning system (TCS-SP2, Leica Microsystem, Wetzlar, Germany) and an oil-immersion objective (N.A. 1.4; physical pixel size: 233 nm). For some images, additional 2 ×  magnification was applied. Fluorescent dyes were excited with Ar/ArKr laser (for 488 nm) or HeNe lasers (for 543 and 633 nm). DAPI staining was imaged after two-photon excitation with an ultrafast, tunable, mode-locked titanium:sapphire laser (Chamaleon, Coherent Inc., Santa Clara, CA). All experiments were repeated at least 3 times, and at least 10 randomly chosen microscopic fields were analyzed for each condition. Immunoreactivity for pGSK3α/β, pAPP, Aβ42-C-terminus, 4G8 and pCREB was quantified by drawing regions of interest (ROIs) in the acquired field and expressing fluorescence as the sum of intensities (8-bit depth; 256 levels) measured for every pixel in the ROI. Immunofluorescence for synaptophysin and synapsin-1 were quantified as “density” that is the total fluorescence intensity of synaptophysin/synapsin-1 labeling (quantified as above) divided by the total area in the field that was occupied by neurons (identified by MAP2 immunoreactivity and calculated by ImageJ software [available at http://rsbweb.nih.gov/ij/]). In every studied condition, negative controls were obtained by omitting the primary antibody. The operator was blinded to the study conditions.

### Western Blot

Infected or mock-infected mouse cortical neurons (12-15 DIV) were washed twice with PBS and scraped in cold RIPA buffer containing 1 mM phenylmethylsulfonyl fluoride, phosphatase and protease inhibitor mixtures (Sigma), and 0,1% sodium dodecyl sulfate. After incubation for 30 min on ice, cellular suspensions were centrifuged (10,000 × g for 30 min at 4 °C) and the supernatants were collected and assayed to determine their protein concentration (Micro BCA protein assay, Thermo Fisher Scientific, Waltham, MA). Equivalent amounts of proteins were loaded onto either 8% tris-glycine polyacrylamide gels or 10–20% Novex Tricine precast gels (Invitrogen) for electrophoretic separation, and then electroblotted onto nitrocellulose membranes for Western blot analysis. Membranes were blocked with 5% nonfat dry milk in tris-buffered saline containing 0.1% Tween-20 for 1 h at RT and incubated with primary antibodies (rabbit polyclonal anti-GSK-3β, Cell Signaling; rabbit polyclonal anti-pGSK3 α/β [Tyr279/Tyr216], Signalway antibody; rabbit polyclonal anti-pGSK3 αβ [Ser9] Cell Signaling; rabbit polyclonal anti APP, Cell Signaling; rabbit polyclonal anti pAPP [Thr668], Cell Signaling; rabbit polyclonal anti synapsin-1 XP, Cell Signaling; mouse monoclonal anti synapthophysin, Immunological Science; mouse monoclonal anti Beta-Amyloid, 17–24 [4G8], Covance; mouse monoclonal anti-αTubulin, Sigma; mouse monoclonal anti-GAPDH, Abcam) at a final concentration of 1 μg/mL. After incubation with appropriate secondary horseradish peroxidase-conjugated antibodies (1:2,000; Cell Signaling), visualization was performed with ECL plus (GE Healthcare, Amersham Place, Buckinghamshire) using either UVItec Cambridge Alliance or Kodak X-OMAT films. In the latter case,images of the blots were acquired using a high-resolution scanner. When necessary, membranes were stripped by heating at 56 °C in 62.5 mM Tris-HCl, pH 6.7, with 100 mM 2-mercaptoethanol and 2% SDS and re-probed. Molecular weights for immunoblot analysis were determined using BenchMark™ Pre-Stained Protein Ladder (Invitrogen). Densitometric analysis was carried out with Image J software. Experiments were repeated at least 3 times.

### Electrophysiological measurements in autaptic microcultures

Autaptic cultures of cortical neurons were prepared as previously described^23^,^24^. Glass coverslips coated with agarose, which does not allow cell growth, were sprayed with a mixture of poly-D-lysine and collagen (both from Sigma) to create microislands where glial cells could be grown. Cortical astrocytes from the brains of postnatal day 0-to-2 C57/Bl6 mice were cultured according to standard procedure. Dissociated cells (suspended in Dulbecco’s MEM supplemented with 10% fetal bovine serum and antibiotics) were plated onto the coverslips. After 4–6 days, when glial microislands had formed, half the medium volume was replaced with neuronal medium (consisting of Neurobasal medium, 2% B-27, 0.5% glutamine, and 1% PSN). Cortical neurons from postnatal day 0-to-2 C57/Bl6 mouse brains were prepared according to standard procedure^5^ and suspended in neuronal medium. Neurons were then plated onto glial microislands at low density (25 × 10^3^/cm^2^) to obtain a ratio of one neuron per island. Every 4 days half the neuronal medium volume was replaced with fresh neuronal medium supplemented with 2 μM cytosine arabinoside. Autapses were studied from 9 to 21 DIV.

Synaptic transmission was studied using the patch-clamp technique in the whole-cell configuration as previously described^24^,^59^,^60^. An Axopatch 200B amplifier (Molecular Devices, Sunnyvale, CA) was used, and stimulation and data acquisition were performed with the Digidata 1200 series interface and pCLAMP 10 software (Molecular Devices). Recording electrodes were fabricated from borosilicate glass capillaries with the aid of a micropipette puller (P-97, Sutter Instruments, Novato, CA). The extracellular solution (pH 7.4) contained (in mM): 140 NaCl; 2 KCl; 10 HEPES; 10 glucose; 4 MgCl_2_; and 4 CaCl_2_. The internal solution (pH 7.4) contained (in mM): 136 KCl, 17.8 HEPES, 1 EGTA, 0.6 MgCl_2_, 4 ATP, 0.3 GTP, 12 creatine phosphate, and 50 U/ml phosphocreatine kinase. Neurons were voltage-clamped at a membrane potential of −70 mV, and stimuli mimicking action potentials (2 ms at 0 mV) were delivered to evoke EPSCs, which were recorded every 6 or 20 s. Input resistance, access resistance, and membrane capacity were monitored before and at the end of the experiments to ensure recording stability and the health of studied cells. The amplitudes and frequency of spontaneous mEPSCs were evaluated in 60-s recordings. The detection threshold was set to 3.5 times the baseline standard deviation.

### Statistics

Statistical comparisons were performed with Systat 10.2 software. All data were expressed as mean ± standard error of the mean (SEM) and followed normal distribution, as assessed by the same software. In order to asses whether HSV-1 infection significantly affected the measured parameters, the two-tailed Student’s *t* test was used. One-way ANOVA with Bonferroni’s post-hoc test was used for multiple-comparisons. For experiments that included fewer than 10 observations (e.g. densitometric analysis of WB data), the Mann-Whitney (Wilcoxon) statistic was used. The level of significance was set at 0.05.

### Ethics Statement

All methods were carried out in accordance with the guidelines approved by the Ethics Committee of the Catholic University. All experimental and animal procedures were fully compliant with Italian (Ministry of Health guidelines, Legislative Decree No. 116/1992) and European Union (Directive No. 86/609/EEC) legislation on animal research. Efforts were made to limit the number of animals used and to minimize their suffering.

## Additional Information

**How to cite this article**: Piacentini, R. *et al.* Herpes Simplex Virus type-1 infection induces synaptic dysfunction in cultured cortical neurons via GSK-3 activation and intraneuronal amyloid-β protein accumulation. *Sci. Rep.* **5**, 15444; doi: 10.1038/srep15444 (2015).

## Supplementary Material

Supplementary Information
