# Supplementary material for: Herpes Simplex Virus type-1 infection induces synaptic dysfunction in cultured cortical neurons via GSK-3 activation and intraneuronal amyloid-β protein accumulation
Source: Sci Rep. 2015 Oct 21;5:15444. doi: 10.1038/srep15444 (PMC4614347; doi:10.1038/srep15444)
Supplement: Supplementary Information [file srep15444-s1.pdf]

Herpes Simplex Virus type-1 infection induces synaptic dysfunction in cultured cortical neurons via GSK-3 activation and intraneuronal amyloid-β protein accumulation

Roberto Piacentini[1#], Domenica Donatella Li Puma[1#], Cristian Ripoli[1], Maria Elena Marcocci[2], Giovanna De Chiara[3], Enrico Garaci[4], Anna Teresa Palamara[4,5], Claudio Grassi[1,4]*

[1] Institute of Human Physiology, Medical School, Università Cattolica, 00168 Rome, Italy

[2] Department of Public Health and Infectious Diseases, Sapienza University of Rome, 00185 Rome, Italy

[3] Institute of Translational Pharmacology, National Research Council, 00133 Rome, Italy

[4] San Raffaele Pisana Scientific Institute for Research, Hospitalization and Health Care, 00163 Rome, Italy

[5] Department of Public Health and Infectious Diseases, Institute Pasteur Cenci Bolognetti Foundation, Sapienza University of Rome, 00185 Rome, Italy

[#]R.P. and D.D.L. contributed equally to this work.

*Corresponding Author: Claudio Grassi, Institute of Human Physiology, Università Cattolica, Largo Francesco Vito 1, 00168 Rome, Italy. E-mail: grassi@rm.unicatt.it. Phone: +39-06-3015-4966; Fax: +39-06-3015-4665

Figure legends

Suppl. Figure 1: HSV-1 induces phosphorylation of GSK-3 at Ser9/21.

(a-b) Representative examples of mock- and HSV-1-infected neurons at 18 h p.i. immunostained for $pGSK^{S9/21}$ (green) and MAP2 (red). **(c)** Bar graphs showing mean fluorescence intensities of GSK phosphorylation at Ser9/21 in the conditions represented in a-b. (**d**) Western blot analysis of $pGSK\text{-}3^{S9/21}$, total GSK-3α/β and GAPDH carried out on lysates of mock- (left lane) and HSV-1-infected (right lane) cells at 18 h p.i.. **(i)** Densitometric analysis of three independent Western blot experiments. Scale bars: 20 µm. *P<0.05 **P<0.001 *vs.* mock.

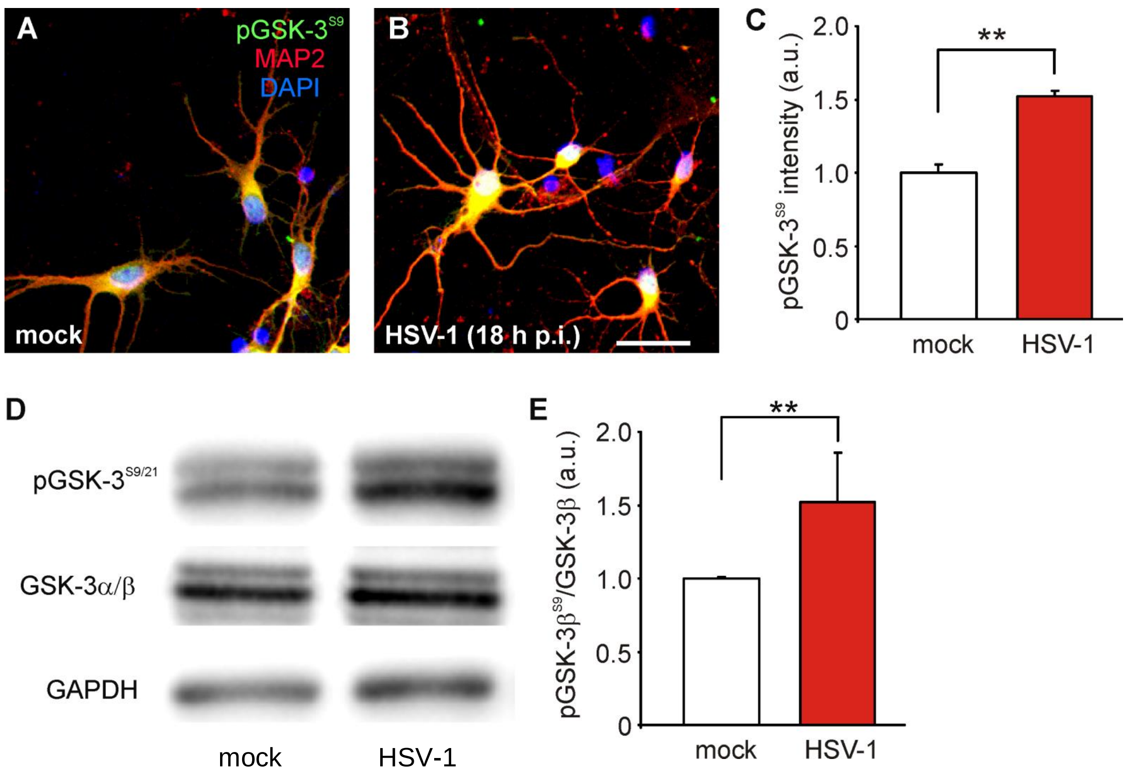
A
pGSK-3S9
MAP2
DAPI
mock
B
HSV-1 (18 h p.i.)
C
2.0
1.5
1.0
0.5
0
pGSK-3S9 intensity (a.u.)
**
mock
HSV-1
D
pGSK-3S9/21
GSK-3α/β
GAPDH
mock
HSV-1
E
2.0
1.5
1.0
0.5
0
pGSK-3βS9/GSK-3β (a.u.)
**
mock
HSV-1
